# Outcomes and treatment for disseminated nocardiosis caused by *Nocardia otitidiscaviarum*: a systematic review

**DOI:** 10.1186/s12879-026-13303-9

**Published:** 2026-05-16

**Authors:** Yazeed Alekrish, Mohammed Alotaibi, Mazin Barry

**Affiliations:** 1 Department of Medicine, College of Medicine, King Saud University, Riyadh, Saudi Arabia; 2 Infectious Diseases Unit, Department of Internal Medicine, College of Medicine, King Saud University, Riyadh, Saudi Arabia; 3 Division of Infectious Diseases, Faculty of Medicine, University of Ottawa, Ottawa, Canada

**Keywords:** Nocardiosis, Disseminated infections, Antimicrobial resistance, *Nocardia otitidiscaviarum*

## Abstract

**Introduction:**

Nocardiosis is a rare aerobic gram-positive cause of infection, often in immunocompromised individuals. Nocardiosis is usually a localized pulmonary disease, however, it can lead to disseminated disease. More than 40 species of *Nocardia* have been described to cause infections in humans, with many having variable susceptibility and resistance patterns to antimicrobials, such as *Nocardia otitidiscaviarum*. Because disseminated infection due to N. otitidiscaviarum is uncommon and has been reported to exhibit distinct and sometimes unpredictable antimicrobial susceptibility, we focused the review specifically on disseminated disease caused by this species.

**Methods:**

A comprehensive and systematic search strategy was developed and included online databases from PubMed, Web of Science, and ScienceDirect to extract all relevant articles published until December 2023. This review has been conducted in line with the PRISMA guidelines. The protocol of this review is registered in the National Institute of Health Research Prospective Register of Systematic Reviews PROSPERO (CRD42024503651).

**Results:**

A total of nine cases of disseminated *Nocardia otitidiscaviarum* were identified using our search strategy. Mean age of patients was 39 years, half of them had a pulmonary initial site of infection, and the other half had skin as an initial site of infection, with the brain being the most common dissemination site. Most patients were immunocompromised, except one case where disseminated infection was reported in an immunocompetent host. The most sensitive antimicrobial agent was amikacin, which was sensitive in all cases; followed by TMP-SMX, and linezolid.

**Conclusion:**

Disseminated *Nocardia otitidiscaviarum* infections are difficult to treat, with poor outcomes, and scarce evidence guiding the best therapeutic approach; this review makes a step in the right direction of shedding some light on the possible therapeutic agents to be used, and their relevant susceptibility and resistance rates, it demonstrates that amikacin has to be included in any patient diagnosed with disseminated *Nocardia otitidiscaviarum*.

**Clinical trial:**

Not applicable.

## Introduction

Nocardiosis is a rare bacterial infection caused by aerobic gram-positive actinomycetes of the genus *Nocardia*. It is weakly acid-fast, branching gram-positive bacilli and is ubiquitous in the environment. However, it is a rare cause of infection, often infecting immunocompromised individuals, causing disseminated disease, especially to the skin, lungs, and brain. Disseminated nocardiosis is defined as the involvement of two noncontiguous sites; it may or may not include a pulmonary focus (the most common site of nocardiosis). In recent years, the incidence of nocardiosis has been on the rise; this is hypothesized to be a result of the increase in the number of immunocompromised individuals [1–4].

As reported by a Canadian study, the yearly incidence of *Nocardia* sp. infections increased from 0.33 to 0.87 per 100,000 individuals over a decade (p-value = 0.001) [5]. Nocardiosis has been reported to be more prevalent in some regions of the world, such as India, Iran, Spain, Pakistan, and Canada [6].

There are more than 40 species of *Nocardia* that have been described to cause infections in humans, the most common species are *Nocardia nova* complex *Nocardia asteroides (N. asteroides)*,* Nocardia brasiliensis (N. brasiliensis)*,* Nocardia farcinica (N. farcinica)*,* Nocardia cyriacigeorgica*, and *Nocardia abscessus* [7]. In addition, the most common form of nocardiosis in immunocompetent individuals is cutaneous nocardiosis; in contrast, disseminated nocardiosis primarily affects the immunocompromised, specifically patients who have impaired cell-mediated immunity [2, 7].

*Nocardia otitidiscaviarum (N. otitidiscaviarum)* (formerly *Nocardia caviae*) was first described to infect the middle ears of guinea pigs in 1924 and was first described in humans with pulmonary infection in 1974 [8, 9]. It is considered a rare and under-reported species of *Nocardia.* that is abundant in nature and reported to have been isolated from different environmental samples including vegetation, soil, many organic matters, in addition to salt-and fresh-water bodies. However, a survey of soil samples in India found that *N. asteroides*, and *N. brasiliensis* were more commonly isolated than *N. otitidiscaviarum* [10, 11].

*Nocardia* sp. have been shown to have different patterns of resistance and susceptibility to antimicrobial therapy, while trimethoprim-sulfamethoxazole (TMP/SMX) is considered the drug of choice for all species, an in vitro study conducted on *Nocardia* sp. isolates found that *N. otitidiscaviarum* and *N*,* farcinica Complex* had 27% and 8% resistance rate to TMP/SMX, respectively [12]. More commonly, resistance to imipenem-cilastatin, which is often considered as second line therapy, is frequently encountered. These findings may suggest that different treatment strategies are warranted for *N. otitidiscaviarum* [13].

The aim of this study was to conduct a systematic review to identify different treatment modalities, resistance rates, and clinical outcomes in disseminated nocardiosis caused by *N. otitidiscaviarum*.

## Methods & materials

### Objectives

The main goal of this systematic review is to provide a comprehensive overview of the outcomes and treatment strategies employed when treating disseminated nocardiosis caused by *Nocardia otitidiscaviarum*.

### Registration

This systematic review has been conducted in accordance with the Preferred Reporting Items for Systematic Reviews and Meta-Analyses (PRISMA) updated guidelines [14]. The protocol of this review is registered in the National Institute of Health Research Prospective Register of Systematic Reviews PROSPERO (CRD42024503651).

### Literature search strategy

A comprehensive and systematic search strategy was developed including online databases from MEDLINE/PubMed, Web of Science, and ScienceDirect to extract all relevant articles published until December 2023 in these online databases. The search strategy incorporated both text words and medical subject heading (MeSH) terms. Key terms such as; (“Nocardia otitidiscaviarum” OR otitidiscaviarum) AND (“disseminated nocardiosis” OR “systemic nocardiosis” OR “extrapulmonary nocardiosis”) were used during the search strategy. The databases were searched from inception until December 2023 without any time limitations.

### Screening and selection of studies

Database search results were extracted into Rayyan.ai online software to facilitate the screening process [15]. The titles and abstracts of extracted studies were reviewed by two authors to assess their eligibility for inclusion based on pre-set inclusion and exclusion criteria. Any disagreements were discussed and resolved with a senior author. The second phase was full-text article screening of studies that passed the first phase of screening. Two authors read the full-texts of these articles to ensure the application of the inclusion criteria. Any disagreements were discussed with a senior author. The screening process was documented using PRISMA flowchart.

### Data extraction

Using a pre-designed data extraction sheet, one reviewer extracted the data of interest from each included study using this data extraction form. After completion, a second independent author reviewed the extracted data for any inconsistencies. Any inconsistency was discussed with a senior author for clarification.

The following information was extracted from included studies:

1. Study characteristics: author, year of publication, country, study design, sample size, follow-up duration, strain of *Nocardia* causing the infection, and inclusion/exclusion criteria.
2. Patient characteristics: Include age, sex, key medical history, and risk factors, such as immunosuppression/immunocompromised states, steroid use, malignancy, or transplant. Also includes medication history, sites of *Nocardia* dissemination, and identify the strain of *Nocardia*.)
3. Intervention characteristics: Type of antimicrobials used, susceptibility patterns, timing of administration, and mode of delivery.
4. Outcome measures: Survival, adverse events, complications, length of hospital stay.
5. Data analysis: statistical methods used, effect size, confidence intervals, heterogeneity, and publication bias.
6. Conclusion: the study’s main findings, limitations, and recommendations for future research.

### Inclusion and exclusion criteria

In this systematic review, we used a pre-set inclusion and exclusion criteria to facilitate the inclusion of all relevant articles and cases, the criteria used were the following:

#### Inclusion criteria

- Studies published without time frame limitations were included in the review.
- Studies that reported the strain of *Nocardia* causing disseminated nocardiosis to be *Nocardia otitidiscaviarum*.
- Only studies published in the English language were included.
- The review only included case reports and case series.
- The review included studies with adult patients.
- The included studies reported the outcomes of interest relevant to the objectives of the review.

#### Exclusion criteria

- Studies published in languages other than English.
- Studies that did not report outcomes of interest for the objectives of the review.
- Studies that included patients under 18 years old.
- Studies that did not report the strain of *Nocardia* causing disseminated nocardiosis or the strain was reported to be any strain other than *Nocardia otitidiscaviarum*.
- Systematic reviews, meta-analyses, narrative reviews, and editorials were excluded.
- Duplicate studies were excluded as well.

### Quality assessment and risk of bias

For quality assessment, two independent authors assessed each included article. We used the critical appraisal checklist from the Joanna Briggs Institute (JBI) for case reports to assess the quality and risk of bias. This tool assesses eight elements in each study, such as, the clarity of patient demographics, clinical condition, medical history, diagnostic and treatment procedures, post-intervention conditions, adverse events, and lessons that can be learned. Each item is scored as ‘Yes’, ‘No’, ‘Unclear’, or ‘Not applicable’ based on the availability of the given information in the article [16].

### Data analysis

We have conducted a qualitative and descriptive synthesis of findings from the included studies. The results of each study were narratively described in terms of baseline demographic characteristics of the included studies; we also compared and recognized trends among included studies in terms of past medical history, treatment strategies used, common dissemination sites, and clinical outcomes. The resistance frequency from all cases have been combined into a table (Table 1) to better demonstrate the resistance patterns of such infections. However, due to the type and number of studies, it was not feasible to do a meta-analysis, we believe that this narrative synthesis and description of the state of literature provides a comprehensive overview of the current state of treatment strategies and outcomes for disseminated nocardiosis caused by *Nocardia otitidiscaviarum*.

**Table 1:** Antibiotic resistance patterns and frequencies from the included cases

Antibiotic	Resistance Cases	Percentage
Amoxicillin-clavulanic acid	5/7	71.4%
Cefepime	4/7	57.1%
Imipenem	4/7	57.1%
Clarithromycin	4/7	57.1%
Ciprofloxacin	3/7	42.9%
TMP-SMX	2/7	28.6%
Tobramycin	2/7	28.6%
Cefoxitin	1/7	14.3%

## Results

### The primary results of the search

**Fig. 1:**
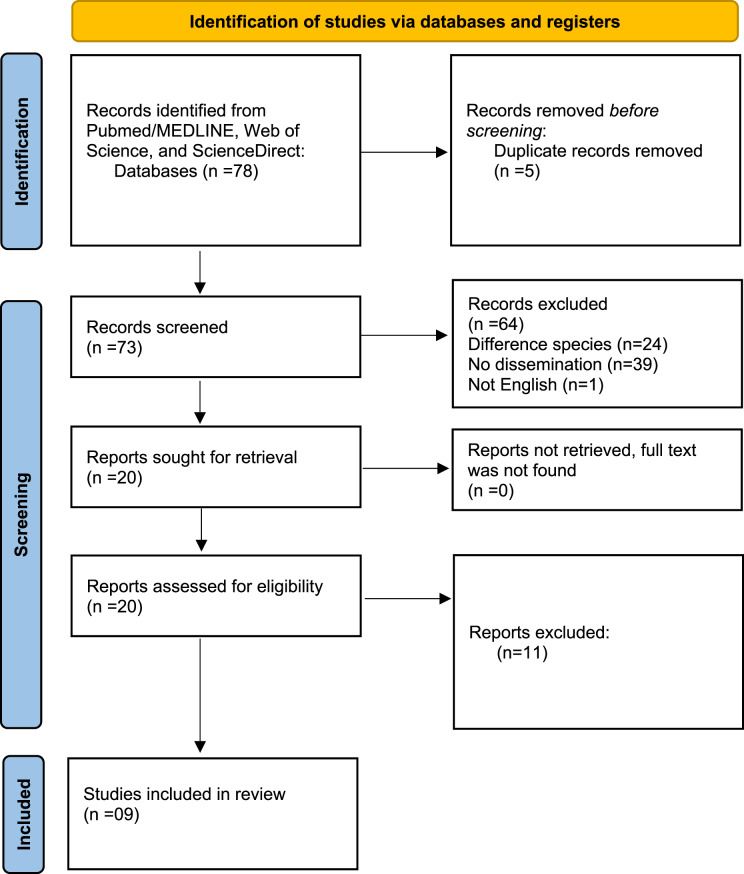
The flowchart of the reviewed studies according to PRISMA

We have found a total of 78 studies through the online database (MEDLINE, Web of Science, and ScienceDirect) search conducted. After that, the studies were imported into Rayyan.ai software, and the process of duplicate detection and removal was initiated. A total of five duplicates were identified and removed. 73 studies remained for the screening process. A two-step screening process was done, the initial phase included screening of the titles and abstracts of the included studies by two independent authors. After that, the second phase of screening involved screening of the full-text articles of studies that passed the first phase. After the screening process was finished; eight case reports were identified and included in the review [17–24]. In addition to these case reports; one retrospective study that included 14 case reports from 1991 to 2002 for nocardiosis was also screened, from these cases in the study, one case fulfilled our inclusion criteria and was also included in our review for a total of nine cases [25]. Our search strategy had no timeframe limitations, and all databases were searched from inception until December 2023; however, all of the selected studies were published between 2004 and 2022 only. The PRISMA flowchart depicting the identification and screening process is detailed in Fig. 1.

### Quality assessment and risk of bias

The quality assessment for the included studies was done using the JBI critical appraisal tool [16]. The assessment showed that on average, all included studies met al.l eight criteria except for the ‘identification and description of adverse or unanticipated events’, this item was only reported in two studies by Duangprasert et al. and Sharma et al. [19, 24]. And across all studies, none of the items received a ‘Unclear’ or ‘Not applicable’ assessment (Table 2).

**Table 2:** Quality assessment of the included studies through the critical appraisal checklist for case reports by Joanna Briggs Institute (JBI)

Criterion	Duangprasert et al. 2022 [24]	Barry et al. 2022 [22]	Kullab et al. 2022 [23]	Parengal et al. 2021 [18]	Talon et al. 2021 [17]	Zheng 2019 [20]	Jiang et al. 2016 [21]	Sharma et al. 2007 [19]	Hemmersbach-Miller et al. 2004 [25]
Q1	+	+	+	+	+	+	+	+	+
Q2	+	+	+	+	+	+	+	+	+
Q3	+	+	+	+	+	+	+	+	+
Q4	+	+	+	+	+	+	+	+	+
Q5	+	+	+	+	+	+	+	+	+
Q6	+	+	+	+	+	+	+	+	+
Q7	+	-	-	-	-	-	-	+	-
Q8	+	+	+	+	+	+	+	+	+

Q1, Were details about the patient’s demographic characteristics thoroughly provided?; Q2: Was the patient’s medical history clearly laid out in chronological order?; Q3, Was the patient’s clinical condition upon presentation detailed clearly?; Q4, Were the diagnostic tests or methods used, along with their results, described comprehensively?; Q5, Were the interventions or treatment procedures explained in detail?; Q6, Was the patient’s condition after the intervention detailed clearly?; Q7, Were any adverse or unanticipated events reported and described?; Q8, Does the case report include lessons that can be learned from the case?

+, Yes; -, No; ?, Unclear

### An overview of the reviewed studies’ characteristics

#### Baseline characteristics

The included studies were from various geographical locations, including two cases from Saudi Arabia and one case from Thailand, Qatar, Singapore, China, the United States, and Spain. The ages of included patients ranged from 29 to 59 years, with a mean age of 39 years. Three males and five females were reported in the eight case reports. Various comorbidities were described in the reported patients including autoimmune hepatitis, metastatic breast cancer, systemic lupus erythematosus (SLE) with multi-organ involvement, chronic kidney disease (CKD), and diabetes mellitus (DM). The clinical presentation was not uniform, as the symptoms ranged from fever and seizures, to cough, body aches, skin rashes, and more severe presentations such as bilateral anterior cerebral infarction. Half the cases reported the lungs to be the primary site of infection, and the other half reported the skin being the primary site, with the brain being the most common dissemination site [17–25].

#### Diagnosis

In regard to diagnostic methods, the identification of *Nocardia* typically involved various culture methods, primarily from blood samples augmented by advanced techniques such as matrix-assisted laser desorption/ionization-time of flight mass spectrometry (MALDI-TOF MS) for precise organism identification. In the majority of studies, staining methods included gram stain, acid-fast staining, and modified Kinyoun stain for the initial identification, which in some cases revealed, gram-positive branching filamentous and partially acid-fast bacilli (A detailed summary of baseline characteristics of the included studies is presented in Table 3).

**Table 3:** Summary of baseline characteristics, clinical features, and diagnostic criteria for disseminated Nocardia otitidiscaviarum infections in the included studies

Study and Study Design	Geographical location	Sample and Demographics	Clinical Presentation	Co-morbidities	Diagnostic Techniques	Staining Results	Verification via MALDI-TOF	Sites of Infection
Duangprasert et al. 2022; Case report [24]	Thailand	*n* = 1; age 51 years; male	Fever, bilateral anterior cerebral infarction	Autoimmune hepatitis	Pus culture	Gram and acid-fast stains showed Gram-positive branching filaments	Yes	Lungs, brain
Barry et al. 2022; Case report [22]	Saudi Arabia	*n* = 1; age 37 years; female	Seizure and multiple skin nodules	Metastatic breast cancer (Stage IV)	skin and brain tissue	modified Kinyoun stain positive	Yes	Skin, lungs, brain
Kullab et al. 2022; Case report [23]	Saudi Arabia	*n* = 1; age 36 years; female	Fever	Metastatic breast cancer (Stage IV)	Culture of multiple tissues	not reported	Yes	Skin, brain
Parengal et al. 2021; Case report [18]	Qatar	*n* = 1; age 29 years; female	Fever, dry cough, SOB, body aches skin rash, and gastric irritation	Systemic SLE with multi-organ involvement	Culture of sputum, BAL, and skin pustules	Gram positive staining showed branching filaments of gram-positive bacilli. Modified Ziehl–Neelsen Staining showed partially acid-fast bacilli.	Yes	Lung, brain, liver, spleen, and kidneys
Talon et al. 2021; Case report [17]	not reported	*n* = 1; age 30 years; female	Jaundice, bloating, and diarrhea	Hepatic impairment	BAL culture	Not reported	Not reported	Liver, lungs, brain
Zheng 2019; Case report [20]	Singapore	*n* = 1; age 59 years; male	Fever and painful soft tissue swellings	HTN, Dyslipidemia, stage Ⅳ CKD	Blood culture	Negative for Mycobacterium tuberculosis	Not reported	Lungs, skin
Jiang et al. 2016; Case report [21]	China	*n* = 1; age 37 years; male	Cough with sputum	Subclinical hypothyroidism	Culture of cervical lymph node with modified acid‑fast stain	Acid-fast positive organism with smooth and granular colonies	Not reported	Lungs, skin, liver
Sharma et al. 2007; Case report [19]	United States	*n* = 1; age 36 years; female	Fever	Sickle cell anemia, ESRD, DCM, HTN	Blood culture	Showed positive findings with modified acid-fast staining	Not reported	Lungs, blood
Hemmersbach-Miller et al. 2004; Case report [25]	Spain	*n* = 1	Purulent expectoration (71.4%), fever (50%), and dyspnea (42.9%)	COPD, Renal transplant, DM	Culture of multiple tissues	not reported	Not reported	Lungs, skin, brain

BAL, Bronchoalveolar Lavage; COPD, Chronic Obstructive Pulmonary Disease; CKD, Chronic Kidney Disease; DCM, Dilated Cardiomyopathy; DM, Diabetes Mellitus; ESRD, End-Stage Renal Disease; HIV, Human Immunodeficiency Virus; HTN, Hypertension; MALDI-TOF, Matrix-Assisted Laser Desorption/Ionization-Time of Flight; SLE, Systemic Lupus Erythematosus

### Resistance and susceptibility patterns

Antibiotic susceptibility testing was carried out by microdilution and disk diffusion methods in the majority of the included studies, only two cases did not report any susceptibility results.

Among the cases that reported susceptibility results, the most effective agent was amikacin, demonstrating susceptibility in all 7 of the 7 cases. This was followed by TMP-SMX and linezolid, both showing susceptibility in 5 out of 7 cases, and ciprofloxacin, which was susceptible in 3 out of 7 cases. The susceptibility and resistance results were summarized in Tables 1 and 4.

**Table 4:** Antibiotic susceptibility patterns and frequency from the included cases

Antibiotic	Susceptible Cases	Percentage
Amikacin	7/7	100%
TMP-SMX	5/7	71.4%
Linezolid	5/7	71.4%
Ciprofloxacin	3/7	42.9%
Moxifloxacin	2/7	28.6%
Tobramycin	2/7	28.6%
Gentamicin	2/7	28.6%
Minocycline	1/7	14.3%

### Management and treatment strategies

Multiple antibiotic regimens were utilized in the management of disseminated *Nocardia otitidiscaviarum* in the reported cases. Four of the included studies also included individualized surgical interventions in the management. As reported in Duangprasert et al., they utilized bifrontal decompressive craniectomy for aneurysm obliteration [24], Hemmerbach-Miller et al. reported surgical drainage of multiple intracranial abscesses [25], Talon et al., case with hydrocephalus, was managed with an external ventricular drain [17], Barry et al. case underwent a left parietal mini-craniotomy which drained frank pus and necrotic tissue, with histopathological features consistent with brain abscess and no granulomas [22].

In terms of antimicrobial management of the infection, multiple regimens were used across different studies, such as in Duangprasert et al. who utilized a regimen consisting of TMP-SMX, amikacin, and moxifloxacin [24]; which was almost similar to the antimicrobial regimen employed in Barry et al., where they utilized a combination of imipenem-cilastatin, TMP-SMX, and amikacin; however, despite this broad-spectrum management, both patients died eventually [22, 24]. In the case of Hemmersbach-Miller et al., they have used a higher number of antimicrobials including a combination of TMP-SMX, ofloxacin, ciprofloxacin, ceftazidime, and vancomycin over a long period of 147 days; surgical drainage of multiple intracranial abscesses was also performed. However, despite the long-term antimicrobial management, the patient eventually died [25]. In Talon et al., intravenous TMP-SMX was combined with intrathecal amikacin; however, the patient also died [17].

Among the included cases, there were four cases in which complete eradication of the infection was reported. Such as in Parengal et al. in which after the management with intravenous meropenem, TMP-SMX, and amikacin, the patient was switched to oral moxifloxacin and TMP-SMX for a year-long course, after which, the patient achieved complete resolution of brain abscesses; albeit it was with a poor neurological outcome [18].

In another case, Zheng et al. utilized a treatment strategy consisting of initially treating with imipenem and TMP-SMX, then subsequently adding linezolid and tigecycline, followed by maintenance therapy with moxifloxacin and TMP-SMX, which resulted in successful resolution of the infection [20]. Treatment was initiated in Jiang et al. with ceftriaxone and TMP-SMX, later transitioning to TMP-SMX and minocycline, which resulted in the successful resolution of the infection after a four-month long treatment course [21]. In Sharma et al. the infection was successfully eradicated after initiating IV TMP-SMX, and then switching to IV amikacin, oral gatifloxacin, and switching TMP-SMX to oral administration [19].

In only one study by Kullab et al., it was reported that the patient’s condition improved, but the infection was not completely eradicated. The treatment regimen used in this case was a combination of amikacin, linezolid, and ceftriaxone, with the addition of moxifloxacin after five weeks of management [23] (The detailed summary of treatment strategies utilized in the included studies is described in Table 5).

**Table 5:** Summary of treatment strategies, and clinical outcomes for disseminated *Nocardia otitidiscaviarum* infections in the included studies

Study	Testing for Susceptibility and Resistance	Antibiotic Susceptibility and Resistance	Prescribed Antimicrobial and Dosage	Non-pharmacological intervention	Duration of Treatment	Length of Hospital Stay	Treatment Outcome	Conclusion
Duangprasert et al. 2022	Broth microdilution method	Susceptible to TMP-SMX, amikacin, moxifloxacin, and resistant to imipenem	TMP-SMX (15 mg/kg/day), amikacin (1 g/day), and moxifloxacin (400 mg/day).	Bifrontal decompressive craniectomy for aneurysm obliteration	No duration reported	Not reported	Died due to septic shock	Cerebral nocardiosis can lead to ruptured infected aneurysms in immunocompromised patients
Barry et al. 2022	Broth microdilution method	Susceptible to, ciprofloxacin, amikacin and resistant to TMP-SMX, linezolid, imipenem, cefepime, cefoxitin, amoxicillin–clavulanic acid, tobramycin, clarithromycin	Imipenem-cilastatin 500 mg IV every 6 h, TMP–SMX 15 mg/kg/day IV divided every 8 h, and amikacin 30/ kg/day IV.	left parietal mini-craniotomy with draining of frank pus and necrotic tissue	22 days	22 days	Died	There is a need to consider rare Nocardia species in at-risk patients with relevant occupational exposure
Kullab et al. 2022	Not reported	Susceptible to amikacin, linezolid and resistant to TMP-SMX, ciprofloxacin, imipenem, cefepime, amoxicillin-clavulanic, tobramycin, clarithromycin	Amikacin, linezolid, and ceftriaxone while moxifloxacin was added after five weeks.	None	More than nine weeks	More than nine weeks	Improved	Nocardia infection, while rare, is becoming more common among patients undergoing immunosuppressive therapy for hematological conditions or solid tumors
Parengal et al. 2021	Epsilometer testing	Susceptible to TMP-SMX, amikacin, ciprofloxacin, moxifloxacin, linezolid and resistant to amoxicillin-clavulanate, ceftriaxone, clarithromycin	Initiated with IV meropenem (meningeal dose), TMP-SMX, and amikacin; transitioned to oral moxifloxacin and TMP-SMX for one-year course	None	8 weeks of IV antibiotics, then planned for 10 months of oral antibiotics	Not reported	Complete resolution of brain abscesses but poor neurological outcome	Physicians treating SLE patients must recognize the potential for rare infections with atypical presentations
Talon et al. 2021	Not reported	Not reported	IV TMP-SMX and intrathecal amikacin	Drainage of hydrocephalus through an external ventricular drain	No duration reported	Not reported	Died	In patients who exhibit cavitary lung lesions along with brain lesions, it is essential to consider nocardiosis as a potential diagnosis.
Zheng 2019	Broth microdilution method	Susceptible to amikacin, tobramycin, TMP-SMX, linezolid, and resistant to amoxicillin/clavulanic acid, ceftriaxone, cefepime, imipenem, ciprofloxacin, clarithromycin.	After 7 days, started with imipenem and TMP-SMX. After 6 weeks, the regimen includes TMP-SMX, linezolid, and tigecycline. For maintenance therapy, moxifloxacin and TMP-SMX were used.	None	More than 6 weeks	Not reported	Cured	Given the varied resistance patterns across different Nocardia species, it is crucial to early identify the causative species and determine its susceptibility results.
Jiang et al. 2016	Disk diffusion method	Antibiotics with area of inhibition were amoxicillin- clavulanic acid, 6 mm; cefepime 6 mm; imipenem, 6 mm; cefotaxime, 6 mm; ceftriaxone, 9 mm; erythromycin, 12 mm; ampicillin, 16 mm; gentamicin, 22 mm; ciprofloxacin, 24 mm; TMP-SMX, 30 mm; linezolid, 38 mm; minocycline, 40 mm; and amikacin, 42 mm.	Initial treatment includes Ceftriaxone at 2 g/day and TMP-SMX (two tablets twice daily) prior to susceptibility testing. After the susceptibility results, the treatment was switched to TMP-SMX and Minocycline for a duration of 4 months.	None	Four months	Not reported	Cured	Clinical and microbiological manifestations of nocardiosis differ among various Nocardia species; thus, accurate identification of the specific species is essential for effective diagnosis and treatment.
Sharma et al. 2007	Broth microdilution method and disk diffusion method	Susceptible to amikacin, ciprofloxacin, gaitifloxacin, clarithromycin, gentamicin, kanamycin, tobramycin, sulfisoxazole, and linezolid	Treatment started with IV TMP-SMX (320/1600 mg every 24 h), followed by oral administration. The regimen was then changed to IV amikacin 300 mg OD and oral gatifloxacin 200 mg OD for two weeks, continuing with gatifloxacin for seven months.	None	IV amikacin + oral gatifloxacin for 2 weeks, then gatifloxacin for 7 months	More than 14 days	Cured	Combination of sickle cell anemia and ESRD may have synergistically contributed to significant T-cell suppression, predisposing her to disseminated nocardiosis.
Hemmersbach-Miller et al. 2004	Not reported	Not reported	TMP-SMX; ofloxacin; ciprofloxacin; ceftazidime; vancomycin with treatment duration of 147 days.	Surgical intervention for the drainage of multiple intracranial abscesses	147 days	Not reported	Died	Nocardiosis should be considered in the differential diagnosis when patients present with pulmonary symptoms, soft tissue infections, or brain abscesses.

ESRD, End-Stage Renal Disease; IV, Intravenous; TMP-SMX, Trimethoprim-Sulfamethoxazole

## Discussion

### Clinical manifestations and resistance patterns

*Nocardia* sp. infections can cause either local or disseminated infections, local infections most commonly involve the lungs as the primary site. With disseminated infections usually disseminating, most commonly, to the brain and skin [26]. This aligns with our review, in which almost all included cases had pulmonary, brain, and skin involvement.

Local infections are usually easier to treat and have better outcomes than disseminated infections. All *Nocardia* species can cause disseminated infections, however, the best therapeutic approach for each species is not clear, as it was demonstrated that resistance patterns of *Nocardia* varies among different species and in different geographic locations [27]. Although TMP-SMX is one of the most commonly used antimicrobials for nocardiosis, some studies demonstrated high resistance patterns to this antibiotic, such as one study conducted in Thailand which showed that *Nocardia* isolates had a 58% resistance rate to TMP-SMX [28]. *Nocardia otitidiscaviarum* has been reported to have extremely variable resistance rates to TMP-SMX and different antimicrobials, typical in-vitro susceptibility patterns, as per the Manual of Clinical Microbiology, shows it to be susceptible to amikacin, ciprofloxacin, gentamicin, linezolid, and sulfamethoxazole, with resistance to amoxicillin-clavulanic-acid, ceftriaxone, and imipenem [29]. The cases reported in the literature shed some light on the susceptibility patterns of disseminated *Nocardia otitidiscaviarum* in clinical practice. Among the cases included in our review, five out of seven were susceptible to TMP-SMX, however, some cases of localized *Nocardia otitidiscaviarum* showed a high rate of resistance to TMP-SMX, as reported in four cases of non-disseminated *Nocardia otitidiscaviarum* infections [30–33].

These findings highlight the conflicting nature of resistance of this organism to TMP-SMX, which could benefit from a refined empiric treatment strategy to overcome these variable rates of resistance.

### Management strategies and identification methods

All cases reported in the literature utilized a combination of antimicrobials for the management of disseminated *Nocardia otitidiscaviarum.* The main antimicrobial used in almost all cases was TMP-SMX, which is based on common practice and previous literature of in vitro susceptibility testing of *Nocardia* species, showing that TMP-SMX is one of the most favorable antimicrobials for this organism. Other antimicrobials that also showed favorable results in in-vitro testing include amikacin, doxycycline, and minocycline; however, these results varied with different species, but two of the most consistently favorable antibiotics were TMP-SMX and amikacin. Therefore, these agents could be included in most empiric regimens before susceptibility testing and species identification are available [34, 35].

Initial treatment regimens used across published disseminated *N. otitidiscaviarum* cases were heterogeneous and mostly derived from case reports and in vitro susceptibility testing; therefore, guideline-level recommendations cannot be made. However, initial treatment choices can be concluded from these susceptibility testing results. Initial use of amikacin and TMP-SMX are reasonable options given that they have the highest susceptibility rates. The final antimicrobial regimen choice should be based on susceptibility results as soon as they are available.

The use of adjunct surgical management was reported in almost half *Nocardia otitidiscaviarum* cases reported included in this review, this is due to the disseminated nature of the disease, and the decision for surgical interventions was individualized for each case, depending on the presentation, the dissemination site, presence of complications, and the general condition of the patient. These decisions should be considered when patients present with complications requiring surgical interventions, such as hydrocephalus or intracranial abscesses.

The outcomes of dealing with this infection could be improved by the appropriate and speedy utilization of antimicrobials. Since the diagnosis of disseminated nocardiosis can be challenging, both the utilization of appropriate antimicrobials and the rapid identification of the organism are crucial.

The current standard practice of identifying *Nocardia* species is by modified Kinyoun staining, which is a method of acid-fast staining that does not use heat in the process and is better for detecting weakly acid-fast organisms such as *Nocardia*[36, 37]. Then, once the presence of *Nocardia* is established, MALDI-TOF MS is used to determine the species of *Nocardia*, which works by ionizing specific protein molecules of the organism with a laser, then measuring their mass-to-charge ratios, and comparing the result with a reference database for identification of specific organisms [12, 38, 39]. *Nocardia* species are usually cultured initially on routine bacteriologic media such as 5% sheep blood agar, chocolate agar, and BACTEC blood culture broth media. However, when the suspcision of *Nocardia* is high, the addition of selective media usually yields more accurate results, media such as colistin-nalidixic acid agar, modified Thayer-Martin agar, and buffered charcoal-yeast extract (BCYE) agar [26].

Other more modern methods of identification techniques such as Polymerase Chain Reaction-Restriction Fragment Length Polymorphism (PCR-RFLP) amplification, which amplifies a portion of the organisms’ 16 S rRNA gene; this method is specific and accurate. However, it is not widely available and is used mostly for research purposes [40, 41].

### Strengths of the review

As identified in this review very few cases reports disseminated nocardiosis due to *Nocardia otitidiscaviarum* and due to the variable presentations and unclear treatment strategies, it is difficult to conclude robust clinical decisions based on current evidence; However, this review is a step forward in understanding the most commonly used treatment strategies related to this infection and could provide guidance and assistance in making informed clinical decisions, until larger multicenter clinical trials of this form of disseminated disease are performed.

#### Limitations and future directions

Although this review systematically included all the relevant literature regarding disseminated *Nocardia otitidiscaviarum*, the number of cases is low and is not sufficient for any recommendations and guidelines; however, it could provide guidance in making clinical decisions based on the current available evidence. Another limitation of this review is that it included solely case reports, this limits the generalizability of the results and leads to other biases inherent to case reports such as publication bias, and also might suffer from selective/missing data. It is also worth mentioning that some cases did not use MALDI-TOF confirmation or molecular confirmation of the species (as summarized in Table 3) which could lead to some uncertainty regarding the pathogenic species causing the disease. We recommend further research to include more cases of this infection and to compare different treatment regimens head-to-head and compare their outcomes in a more controlled fashion. This will further shed some light and refine the treatment strategies used for this condition.

## Conclusions

The difficulty in diagnosing disseminated infection due to *Nocardia otitidiscaviarum* and the poor outcomes associated with it highlight the need for more refined treatment strategies. This review helps shed some insight on the treatment strategies and outcomes used in the reported literature. We have found some consistent aspects among all cases, such as the presence of an immunosuppressive condition or major comorbidities in most cases, although disseminated infection in an immunocompetent host was also reported in one case. It was also found that the majority of cases were resistant to specific antimicrobials, such as, Amoxicillin-clavulanic acid, cefepime, imipenem, and clarithromycin, on the other hand, some antimicrobials showed more favorable activity against the organism, such as, Amikacin, TMP-SMX, and linezolid. However, even with these antimicrobials, the outcomes of disseminated nocardiosis remain poor, as four out of the nine cases included in our review died, which underscores the need for better and more rapid diagnostics and therapeutic approaches.

## Data Availability

All secondary data used and synthesized in this manuscript will be available upon request from the corresponding author.
